# Factors Influencing the Willingness to Pay for Wetland Bird Protection: A Value Assessment Based on a Questionnaire Survey of Residents in Gahai Wetland, Gansu, China

**DOI:** 10.3390/ani15152183

**Published:** 2025-07-24

**Authors:** Xiushan Li, Xiaoliang Shi, Tiantian Yu, Jinhong Du, Tom D. Breeze

**Affiliations:** 1 Key Laboratory of Southwest China Wildlife Resources Conservation of the Ministry of Education, College of Life Science, China West Normal University, Nanchong 637009, China; xiushanli@vip.163.com; 2 College of Economics and Management, Shenyang Agricultural University, Shenyang 110866, China; 3 State Key Laboratory of Genetic Resources and Evolution, Kunming Institute of Zoology, Chinese Academy of Sciences, Kunming 650203, China; 4 Chinese Research Academy of Environmental Science, Beijing 100012, China; 5 Centre for Agri-Environmental Research, School of Agriculure, Policy & Development, University of Reading, Reading RG6 6AR, UK; t.d.breeze@reading.ac.uk

**Keywords:** birds, wetlands, protected areas, market technology assessment, religious belief

## Abstract

The Gahai wetland in Gansu, China, is a key wetland for nationally and globally threatened bird species. This research aims to quantify the economic value of wetland birds by the method of contingent valuation questionnaire, thereby providing a foundation for conservation of avian species. The result indicated that residents were willing to pay between 208 and 230 CNY (29–32 USD $) annually for bird protection, a total annual value of wetland birds in Luqu County ranging from 7.9 M to 8.8 M CNY (1.1–1.3 M USD $). Men, locals, and older individuals were more inclined to contribute and offered higher payments, albeit non-significant correlation between education level and annual income with the willingness to pay. While the depth of understanding of wetland pressures did not significantly influence the willingness to pay, it did show a positive correlation; those with more profound understanding tended to be more generous in their payments. Tibetan male residents were more likely to prioritize ecological and environmental protection due to their religious beliefs and cultural customs, which emphasize the reverence for and compassion towards birds. This research not only complemented and validated the applicability of the contingent valuation method in China but also underscores the considerations that should be taken into account when employing this method.

## Simple Summary

Wetland birds have immense cultural value in China but are threatened by human activities. We surveyed the views of residents and visitors of the Gahai Wetland system in Tibet and elicited their willingness to pay for the conservation of these wetlands. We found that respondents were willing to pay between 208 and 230 CNY per year to support the conservation of wetland birds in this area. Among the sample, local residents, men (especially ethnically Tibetan men), and older respondents were both more likely to be willing to pay and were willing to pay greater amounts. Our results show that it is important to account for these cultural factors when assessing support for biodiversity conservation efforts.

## 1. Introduction

Wetlands are crucial ecosystems that offer a multitude of ecological services. They provide a spectrum of provisioning services, such as food and fuel [1,2,3], regulating services including water purification and flood regulation [4], as well as carbon sequestration [5]. Additionally, wetlands offer significant cultural services like landscape aesthetics and spiritual sustenance [6,7]. The capacity of wetlands to deliver these services is underpinned by their exceptionally high biodiversity, which is a global characteristic [8,9].

Species richness and abundance of wetland birds are both highly sensitive to, and highly reflective of, changes in the wetland environment such as water pollution and drought [10,11,12,13]. Therefore, wetland birds are key biological indicators in these environments and, research on them contributes to understanding wetland ecology [14], and monitoring the effects of environmental change and human activities on wetlands [15,16]. Many wetland birds were also culturally significant to various peoples throughout East Asia [17,18,19].

In China, wetland ecosystems are particularly rich, hosting approximately 800 species of higher plants, 300 species of birds, and 1000 species of fish. These figures represent 2.8%, 26.1%, and 37.1% of the total species counts for these taxa in the country, respectively [20]. Wetlands play an especially vital role in avian life, serving as habitat for over 200 bird species that are either native to China or migrate through the country along pivotal international waterfowl flyways [21,22,23]. This includes 31 of the 57 endangered waterfowl species found in Asia [24], highlighting the importance of wetlands for the conservation of these vulnerable species.

The Gahai Nature Reserve, located at the ecotone of the Qinghai–Tibet Plateau and the Minshan Mountains, spans the watersheds of the Yellow and Yangtze Rivers, serving as a critical water conservation area and the headwater of the Tao River, a major Yellow River tributary. This internationally recognized wetland (designated a Ramsar Site in 2012 and one of only three in Gansu Province) supports over 21,000 migratory and breeding birds annually [25], including 78 species of conservation concern—a total of 7 protected under the China–Australia Migratory Bird Agreement, 31 under the China–Japan Agreement [26], and 11 listed as threatened in China’s Red List [27]. However, its ecological integrity faces growing threats from overgrazing and unsustainable tourism development, jeopardizing this vital habitat.

Despite the importance of wetlands to biodiversity, the long-term impacts of human activities—such as unreasonable development, reclamation, and dam repair—have often been undertaken without proper valuation of their economic or cultural impacts. This neglect stems partly from the challenge of quantifying ecosystem values, particularly for non-market services like cultural significance or biodiversity conservation.

A case in point is Liang’s [28] valuation of the Gahai Nature Reserve’s ecosystem services at 48.44 billion CNY (6.78 B USD $), including water purification, flood storage, and climate regulation. While such replacement cost methods provide important benchmarks for natural capital accounting, they have inherent limitations: the sensitivity to replacement technology costs, the underestimation of non-use values (e.g., intrinsic species value), and the inability to capture cultural/spiritual values, especially for protected species like wetland birds. This gap motivates our study. We complement Liang’s [29] framework by employing stated preference methods (discrete choice experiments) to quantify the public’s willingness to pay (WTP) for bird conservation—a proxy for valuing these intangible ecosystem benefits. DCEs are a robust non-market valuation approach that allows researchers to quantify the economic value of environmental goods by presenting respondents with trade-offs between different conservation scenarios [30]. Unlike contingent valuation methods, DCEs can reveal preferences for specific attributes (e.g., bird species richness, and water quality) and are less prone to bias, making them particularly suitable for evaluating complex ecosystems like wetlands [31].

Wetland birds serve as an ideal proxy for evaluating the public value of the entire ecosystem, as they are not only critical indicators of the broader ecosystem health but also more visually recognizable and culturally salient than abstract ecological metrics (e.g., biodiversity indices). By measuring WTP for bird conservation, we indirectly capture the value of associated ecosystem services, such as water purification, flood control, and recreational benefits.

Our findings are integrated with Liang’s framework to establish a comprehensive model for quantifying the economic value of wetland services. This combined approach provides policymakers with a more holistic understanding of the trade-offs between conservation efforts and human activities in and around wetlands.

## 2. Materials and Methods

### 2.1. Research Area

Gahai Wetland is located in Luqu County, Gannan Tibetan Autonomous Prefecture, on the eastern edge of Qinghai–Tibet high plateau. Gahai Wetland is located at 102°05′00″–102°29′45″ E, 33°58′12″–34°30′24″ N, and the altitude is 3430–4300 m [32]. Gahai Wetland is located in the source area of the Tao River, an important tributary of the upper Yellow River. It is an important part of the Ruoergai wetland.

The administrative divisions of Gahai Wetland include Gahai Township, Langmusi Town, Larenguan Township, and Gongba Village. The wetland comprises 30 distinct patches with a total area of 57,705.04 hectares [33]. The primary wetland types in this region are permanent rivers, seasonal rivers, permanent freshwater lakes, and swampy meadows. For clarity, we define these wetland types as follows: permanent rivers refer to watercourses that maintain continuous flow throughout the year, sustained by groundwater, snowmelt, or consistent precipitation; seasonal rivers are intermittent watercourses that flow only during specific periods such as rainy seasons or snowmelt periods and may completely dry up at other times; permanent freshwater lakes are standing water bodies that persist year-round with salinity levels below 0.5‰; and swampy meadows are waterlogged areas dominated by herbaceous vegetation with fluctuating water levels. The spatial distribution and areal extent of these wetland types are detailed in Table 1.

We conducted our survey and interviews in and around the Gahai Nature Reserve, a key area for tourists and locals to engage with nature. The reserve hosts more than 20 national and provincial class I and class II protected water birds, including endangered species such as Black-necked Cranes (*Grus nigricollis*) [34,35,36]. There are approximately 11,000 Black-necked Cranes in the world, with China accounting for more than 90% of them [37]. Black-necked Cranes are mainly distributed on the Qinghai–Tibetan High Plateau and swamp areas at an altitude of 3500–5000 m in Gansu and Qinghai province and migrate to the area to breed from the end of March to the beginning of April every year, making it a key habitat for the species.

The survey was conducted in each of the six “regions” including those near to those far away from the reserve: Diebu County, Gahai Township, Luqu County, Hezuo City, Linxia City, and Lanzhou City, from 24 August 2014 to 6 September 2014 (14 days). Ninety percent of respondents were nature reserve residents. In order to analyze the residents’ WTP and the impact of residents in different areas on the WTP, the study also conducted a special survey in Luqu. Extrapolating up, using Luqu County as the evaluation area, according to Luqu County’s Demographic Yearbook, the total population of the county was 38,085.

### 2.2. Questionnaire Design

Before formal data collection, a pilot survey was conducted to refine the study design and test the questionnaire’s feasibility. This pilot involved semi-structured interviews with 20 residents in Luqu County, combining open-ended questions (e.g., ‘What factors influence your support for bird conservation?’) and a draft version of the contingent valuation questionnaire. The open-ended responses revealed that religious beliefs significantly affected residents’ attitudes, while the structured questions confirmed the clarity of payment scenarios. Based on these results, we finalized the questionnaire by adding religious affiliation as a key variable and simplifying ambiguous terms. The questionnaire was split into three parts. The first part was the general descriptive statistics of the respondents such as gender, ethnicity, religion, age, occupation, education level, average annual income, and other information. The second part was the interviewees’ knowledge and subjective evaluation of Gahai Wetland Birds and the wetland ecosystem. Among them, respondents were asked to give a self-reported “degree of understanding” around how wetland damage will affect the survival of birds and their strength of belief that it was “necessary to protect” wetlands and birds. “Degree of understanding” means understanding the importance of birds and wetlands to humans, which is an interdependent and inseparable relationship. “Need to protect” means that birds and wetlands are very important to humans, and if humans want to develop better, they must be well protected.

The third part was to estimate the value of birds. The study first asked the respondents whether they were willing to pay for the conservation of birds in the wetlands. If they were willing, then we further asked them the amount that they would be willing to pay, the reasons for this, and their preferred method of payment.

The survey was designed to be suitable for respondents of different nationalities, occupations and ages, particularly of the diversity of backgrounds and languages of the residents, many of whom are Tibetan. The questionnaire was designed to minimize errors where possible. For example, we conducted a face-to-face interview rather than a postal questionnaire to allow respondents to ask us to clarify questions and ensure validity and high response rate of the questionnaire. Questions were framed in simple language and question takers at the nature reserve spoke both Chinese and Tibetan, in order to translate questions and responses to assist the data collection. Each questionnaire was designed to be completed within 10 min. In terms of payment amount, respondents were first asked whether they were willing to pay for wetland bird protection every year, or if they objected to this payment. They were then asked how much they were willing to pay and then given a payment card to choose when their concept was unclear, to avoid excessive or low payment values as much as possible. However, we acknowledge that face-to-face interviews may introduce social desirability bias, potentially leading respondents to state higher WTP amounts than they would actually pay. Future studies could complement stated preference methods with revealed preference approaches, such as recording real donations, to validate the hypothetical WTP estimates.

In total 332 questionnaires were conducted with 325 valid replies (97.89% recovery rate). An average of 54 people were interviewed in each region. Respondents were mostly local residents, with close economic sources and similar economic capabilities. The sex ratio of the valid sample (N = 325) was 66.15% men and 33.85% women. The questionnaire was divided into six age groups, of which the majority of respondents were 31–40 years old (35.07%), while ≤20 years old (2.46%) and >60 years old (5.23%) accounted for a relatively small number. The age distribution of respondents was quite different. The survey area was mainly dominated by residents near 30 wetland patches.

### 2.3. Analytical Methods for WTP Assessment

To comprehensively assess the determinants influencing the WTP for bird conservation, this study employed two analytical approaches. First, a logistic regression analysis was conducted to ascertain the relative impact of various key respondent factors on the propensity to support bird protection measures. This statistical method allows us to understand not only who is willing to pay but also the factors that make individuals more or less likely to express such willingness. Second, WTP models were utilized to estimate the average monetary value that those willing to pay would offer. These models provide a quantitative estimate of the economic value placed on bird conservation by the respondents.

The rationale for employing dual methodologies stems from the recognition that relying solely on one method in WTP studies can often fall short of capturing the full spectrum of influences on payment willingness. By combining the strengths of both logistic regression and WTP modeling, we aimed to achieve a more nuanced and robust understanding of the economic and attitudinal factors at play. All analyses were performed using SPSS Statistics 26.0 software, ensuring the reliability and accuracy of our statistical inferences.

#### 2.3.1. Logistic Regression

Here, a binary logistic regression model is constructed to explore the factors affecting the likelihood that respondents will express a WTP for bird protection (a binary variable, *Y*, with the values of 1 = payment, 0 = no payment) and other respondent characteristics as independent variables (*X*_1_, *X*_2_, …, *X_n_*)

(1) $$Y=a_{0}+a_{1}X_{1}+a_{2}X_{2}+\ldots +a_{n}X_{n}$$

Wrote *Y* as Logistic:

(2) $$Logit(p)=\ln\left(p/1-p\right)=a_{0}+a_{1}X_{1}+a_{2}X_{2}+\ldots +a_{n}X_{n}$$

#### 2.3.2. Willingness to Pay

This study compares two methods to estimate the economic value of respondents’ WTP for Gahai bird protection. First, we used the median payment per person, multiplied by the proportion of respondents that expressed willingness to pay during the questionnaire analysis [38,39]. This forms the lower limit for our estimates. The second method was to weigh each WTP value stated by the respondents by the % of respondents that expressed that value (Formula (3). This acts as an upper limit.

(3) $$WTP_{m}=\sum_{i=1}^{k}WP_{a_{i}}\frac{n_{i}}{N}$$

In Formula (3), *WTP_m_* was the average WTP of the sample. $WP_{a_{i}}$. was the willingness of residents in the sample to pay at level *i*. N was the total sample size. *n_i_* was the number of people willing to pay $WP_{a_{i}}$ in the sample.

## 3. Results

### 3.1. General Descriptive Statistics

Of the 325 completed questionnaires, 256 respondents were willing to pay for the protection of wetland birds (*WTP_i_* > 0 Yuan/year, accounting for 78.77% of the valid questionnaires. There were 69 respondents who were reluctant to pay for the protection of wetland birds, (*WTP_i_* = 0 Yuan/year), accounting for 21.23%. The WTP for different social and economic factors is shown in Table 2.

Table 2 reveals significant variations in the WTP for bird conservation across different demographic groups. In terms of gender, women exhibited a higher payment rate at 81.82% compared to men at 77.21%. Regarding ethnicity, the payment rate was highest among Tibetans at 97.60%, followed by Han at 62.61% and Hui at 47.62%. The exceptionally high rate among Tibetans may be attributed to the fact that Buddhism, which emphasizes harmony with nature, is a predominant religion in the Tibetan community. In terms of religious affiliation, Buddhists showed the highest payment rate of 97.14%, followed by Muslims at 47.62% and those with no religious affiliation at 61.11%.

Age also played a role, with the highest payment rate observed in the 51–60 years age group at 87.88%, while the lowest was found in the 41–50 years age group at 73.53%. When considering occupation, respondents who were either unemployed (66.67%) or self-employed (57.29%) had higher payment rates compared to those in other professions. Education level significantly influenced WTP, with residents holding a graduate degree or higher (N = 2 with a 100.00% payment rate) being the most willing to pay. Regarding annual income, those earning three to six thousand had the highest payment rate at 100.00%, while individuals earning over 80,000 had the lowest at 0.00%. This suggests a general trend where payment rates decrease as income increases.

While descriptive results suggested differences between ethnic and religious groups (Table 2), the logistic regression indicated these were not statistically significant after controlling for other factors (Tibetan vs. Han: *p* > 0.05; Buddhism vs. no religion: *p* = 0.665). Only gender, age, and perceived necessity of protection showed significant effects (all *p* < 0.05). This implies that apparent group differences may be mediated by these confounding variables.

### 3.2. Comparison of Payment Amount Under Different Attributes

The study compared the annual payment amounts for bird protection among wetland residents with varying attributes. The findings are presented in Table 3.

As can be seen from Table 3, the most common value given across all respondents was 100 < x ≤ 500 CNY (14 < x ≤ 70 USD $) (N = 143; 55.9%) and 59% of respondents were willing to pay a sum >100 CNY (>14 USD $). In terms of gender, men generally expressed higher values than women. Of the three main ethnic groups, the Tibetan respondents expressed higher values than Han or Hui respondents, with 79.8% of respondents being willing to pay > 100 Yuan/annum. Similarly, in terms of religious belief, Buddhist respondents were more likely to express higher WTP than other groups, with 68.2% being willing to pay >100 Yuan/annum. In terms of age, respondents aged between 31 and 40 years were most likely to pay higher values, with 91.1% expressing WTP values > 100 yuan/year. The likelihood of expressing higher WTP then declined with age, although no respondents aged under 20 were willing to pay the higher values. In terms of occupation, excluding soldiers (N = 2), administrative and institutional staff were more likely to have higher WTP values while students mostly expressed lower values. Contrary to expectations, WTP values were not higher among those with higher education or earnings but rather peaked with middle education (76.9% WTP > 100 Yuan/annum) and middle earning. Respondents, with those earning 12,001–24,000 CNY (1674.3–3348.3 USD $) and 24,001–36,000 CNY (3348.4–5022.4 USD $) expressing higher WTP values more often than lower or higher earners. Finally, respondents who did not understand wetlands were more less likely to express higher WTP values than respondents who felt they had a better understanding.

### 3.3. Regression Analysis of Factors Influencing WTP

The results of regression analysis on the influencing factors of WTP are shown in Table 4.

Significance levels were lower than 0.05, which meant that the regression of variables was significant. A negative regression coefficient indicated a negative correlation and vice versa. Gender was significantly negatively correlated in the model, indicating that men were more willing to pay for the protection of birds than women were. Similarly, age and the need to protect are significantly and positively correlated, showing that older respondents and those who believed that protection was necessary were significantly more likely to be willing to pay for bird protection than other respondents.

### 3.4. Evaluation on the Conservation Value of Wetland Birds

Based on the analysis of the influencing factors of Gahai residents’ WTP, we estimated the upper and lower values of WTP.

First, we found the average WTP for the sample according to method 1, as shown in Table 2. From Table 2, the average WTP rate of the sample was 78.77%, the median was 200 Yuan/annum (28 USD $); giving a lower limit WTP of 208.59 Yuan/annum (29.1 USD $). Finally, according to Formula (3) for method 2, the cumulative frequency distribution of the average WTP of the sample was used to estimate the upper limit of WTP, as shown in Table 5, was 230.90 Yuan/annum (32.2 USD $) for the whole sample. Therefore, residents’ WTP for wetland birds was 208.59–230.90 Yuan/annual (29.1–32.2 USD $).

The sample is consistent with the statistical population structure and gender of Luqu County, with the permanent resident population aged 15–59 accounting for 66.56% of the total population. However, the income of respondents is typically higher than the average income for the Tibetan region, which may be related to the small sample area, sample size of the survey and the relative affluence of the area. Based on this, it was estimated that the amount Luqu County was willing to pay for wetland bird protection ranged between 7,944,150.15 and 8,793,826.5 Yuan/annual (1,108,296.3–1,226,835.5 USD $).

## 4. Discussion

Our study reveals exceptionally high WTP for wetland bird conservation among Gahai residents, with average annual values ranging between 208 and 230 CNY (29–32 USD $) and extrapolated county-level values of 7.9–8.8 M CNY (1,102,136.9–1,227,696.8 USD $). These values substantially exceed those reported in similar contingent valuation studies, reflecting the profound ecological and socioeconomic importance of these wetlands to local communities. Three key findings emerge from our analysis.

First, logistic regression identified statistically significant predictors of WTP (*p* < 0.05), with gender (β = −0.592, *p* = 0.002) and age (β = 0.043, *p* = 0.001) showing particularly strong effects. Men demonstrated higher WTP than women, likely reflecting gender roles in this pastoral community where male-dominated occupations like herding (98.65% WTP in our sample) directly depend on wetland resources. Older respondents showed greater support, consistent with patterns of accumulated ecological knowledge and intergenerational stewardship values observed in other agrarian communities [40,41,42,43,44].

Second, the most robust predictor was the perception that protection is “essential” (β = 2.289, *p* = 0.033). While descriptive data showed striking differences among groups, notably Tibetan Buddhists (97.14% WTP) versus non-religious respondents (61.11%), these cultural variables were not statistically significant in our regression model (*p* > 0.05). This suggests that religious and ethnic influences may operate indirectly by shaping protection attitudes rather than directly determining WTP. Future research with larger samples should test this mediation hypothesis through hierarchical modeling.

Third, we found unexpected patterns in socioeconomic factors. Contrary to conventional economic theory, WTP did not consistently increase with income, peaking instead among middle-income groups (3000–36,000 CNY annually). Farmers and herdsmen showed particularly strong support (98.65% WTP), underscoring how direct resource dependence can motivate conservation investment regardless of absolute income levels [45,46,47].

These findings carry important policy implications. (1) Conservation programs should prioritize livelihood-integrated approaches for key demographic groups (e.g., payment schemes for male herders). (2) Messaging should emphasize the necessity of protection, particularly for elders who showed high WTP and may serve as cultural conduits. (3) While cultural values appear influential descriptively, policies should focus on measurable attitudinal drivers like protection necessity until further research clarifies mediation pathways [48,49,50,51].

Several limitations warrant consideration. Our face-to-face survey, while ensuring high response rates, may have introduced social desirability bias [52,53,54,55]. The hypothetical nature of WTP questions means stated values may not perfectly predict actual behavior. Our sample size (N = 325) and geographic focus limit generalizability to urban populations or other ethnic groups. Future studies could employ experimental auctions or longitudinal designs to validate these findings.

Despite these limitations, our results demonstrate substantial economic value placed on wetland conservation by Gahai residents. The integration of these valuation estimates with traditional ecological assessments provides a more comprehensive basis for conservation planning—one that acknowledges both ecological services and community-derived economic values. By focusing interventions on statistically validated demographic and attitudinal predictors while remaining attentive to potential cultural mediators, policymakers can develop more effective strategies for protecting these ecologically and culturally significant wetlands.

## 5. Conclusions

This study provides a comprehensive assessment of residents’ WTP for wetland bird conservation in Luqu County, China, employing rigorous contingent valuation methodology. The findings offer both empirical insights and practical implications for conservation policy in ecologically sensitive regions.

The economic valuation reveals that local residents demonstrate substantial support for wetland conservation, with an average annual WTP ranging between 208 and 231 CNY (29–32 USD $) per person. When extrapolated to the county population, this translates to a total valuation of 7,944,150–8,793,826 CNY (1,108,296.3–1,226,835.5 USD $) annually. These figures not only quantify the economic importance of wetland ecosystems but also provide concrete evidence for policymakers regarding the value local communities place on biodiversity conservation.

Our regression analysis yields particularly noteworthy findings regarding the determinants of WTP. Three factors emerge as statistically significant predictors: gender (*p* = 0.002), with men showing higher WTP than women; age (*p* = 0.001), where older respondents demonstrate greater willingness to contribute; and perceived necessity of protection (*p* = 0.033), indicating that conservation attitudes strongly influence payment decisions. These results challenge some conventional assumptions in environmental economics, particularly regarding socioeconomic factors. Contrary to expectations, neither education level (*p* = 0.487) nor annual income (*p* = 0.220) showed statistically significant effects in our model, suggesting that in this cultural context, demographic and attitudinal factors may outweigh traditional socioeconomic predictors.

The study’s findings regarding cultural and religious factors warrant careful interpretation. While descriptive statistics show striking differences, notably the 97.14% WTP rate among Tibetan Buddhists compared to 61.11% among non-religious respondents-these patterns did not achieve statistical significance in our regression analysis after controlling for other variables. This important distinction suggests that while cultural values may create favorable conditions for conservation support, they may operate through indirect pathways rather than serving as direct determinants of WTP.

These insights carry significant policy implications. First, conservation programs should prioritize engagement with demographic groups showing highest WTP, particularly older male residents. Second, communication strategies should emphasize the essential nature of wetland protection, as this perception proved to be a powerful motivator. Third, while cultural values should be respected and understood, policy should primarily focus on empirically validated predictors rather than assumed cultural influences.

The study acknowledges several limitations that suggest directions for future research. The face-to-face survey methodology, while ensuring high response rates, may have introduced social desirability bias. Additionally, the hypothetical nature of WTP questions means stated values may not perfectly predict actual behavior. Future studies could benefit from mixed-method approaches combining stated preference surveys with revealed preference data, as well as larger sample sizes to better examine potential cultural influences.

## Figures and Tables

**Table 1:** Classification and area distribution of wetland types in Gahai wetland.

Type	Number of Patch (Piece)	Area (%)
Permanent river	13	1675.23 (2.90)
Seasonal river	4	278.52 (0.48)
Permanent freshwater lake	1	4732.30 (8.20)
Swamp meadow	12	51,018.99 (88.42)
Total	30	57,705.04 (100.00)

**Table 2:** Proportion of respondents expressing willingness to pay by different socio-economic demographic factors.

Attribute	Index	Number of Respondents (%)
Gender	Men	215 (77.21)
	Women	110 (81.82)
Nation	Tibetan nationality	167 (97.60)
	Han nationality	115 (62.61)
	Hui nationality	42 (47.62)
	Other nationalities	1 (100.00)
Religion	Buddhism	175 (97.14)
	Islamism	42 (47.62)
	No religion	108 (61.11)
Age	≤20	8 (75.00)
	21–30	85 (83.53)
	31–40	114 (75.44)
	41–50	68 (73.53)
	51–60	33 (87.88)
	>60	17 (82.35)
Occupation	Administrative staff	13 (92.31)
	Employees of institutions	40 (90.00)
	Enterprise/company staff	46 (76.09)
	Individual business	96 (57.29)
	Farmers (herdsmen)	74 (98.65)
	Student	20 (75.00)
	Soldier	2 (100.00)
	Housewife/retirement	28 (85.71)
	No occupation	6 (66.67)
Education level	Primary school and below	156 (82.05)
	Middle school (junior or senior high school)	79 (65.82)
	University (undergraduate, junior college)	88 (84.09)
	Postgraduate and above	2 (100.00)
Average annual income (CNY)	<3000	35 (80.00)
	3000–6000	12 (100.00)
	6001–12,000	32 (90.63)
	12,001–24,000	68 (82.35)
	24,001–36,000	115 (76.52)
	36,001–48,000	45 (66.67)
	48,001–60,000	13 (69.23)
	60,001–80,000	5 (80.00)
	>80,000	0 (0.00)
All respondents	/	325 (78.77)

**Table 3:** Percentage distribution of stated annual WTP amounts by demographic groups (Yuan/annum).

Variable Group	Category	Payment Distribution					Logistic Regression Results	
		0 < x ≤ 50 CNY	50 < x ≤ 100 CNY	100 < x ≤ 500 CNY	500 < x ≤ 1000 CNY	X > 1000 CNY	β (SE)	*p*-Value
All Respondents	/	15.2%	25.4%	55.9%	3.5%	0.3%	−9.641 (2.019)	0.000 ***
Gender	Men	13.3%	24.7%	58.4%	3.6%	0.0%	−0.592 (0.400)	0.002 **
	Women	18.9%	25.6%	51.1%	3.3%	1.1%	-	-
Nation	Tibetan nationality	12.9%	7.4%	74.8%	4.9%	0.0%	-	-
	Han nationality	13.9%	33.3%	50.0%	1.4%	1.4%	-	-
	Hui nationality	40.0%	35.0%	25.0%	0.0%	0.0%	-	-
	Other nationalities	0.0%	0.0%	100.0%	0.0%	0.0%	-	-
Religion	Buddhism	12.4%	19.4%	52.9%	15.3%	0.0%	1.780 (0.708)	0.665
	Islam	40.0%	35.0%	25.0%	0.0%	0.0%	-	-
	No religion	15.2%	36.4%	45.5%	1.5%	1.5%	-	-
Age	≤20	83.3%	16.7%	0.0%	0.0%	0.0%	-	-
	21–30	16.9%	26.8%	50.7%	4.2%	1.4%	-	-
	31–40	8.1%	0.0%	87.2%	4.7%	0.0%	0.043 (0.199)	0.001 ***
	41–50	8.0%	22.0%	68.0%	2.0%	0.0%	-	-
	51–60	20.7%	20.7%	58.6%	0.0%	0.0%	-	-
	>60	35.7%	42.9%	14.3%	7.1%	0.0%	-	-
Occupation	Administrative staff	16.7%	0.0%	83.3%	0.0%	0.0%	-	-
	Employees of institutions	11.1%	8.3%	75.0%	2.8%	2.8%	-	-
	Enterprise/company staff	8.6%	34.3%	48.6%	8.6%	0.0%	-	-
	Individual business	18.2%	25.5%	52.7%	3.6%	0.0%	-	-
	Farmers (herdsmen)	9.6%	27.4%	60.3%	2.7%	0.0%	-	-
	Student	40.0%	46.7%	13.3%	0.0%	0.0%	-	-
	Soldier	0.0%	0.0%	100.0%	0.0%	0.0%	-	-
	Housewife/retirement	20.8%	25.0%	50.0%	4.2%	0.0%	-	-
	No occupation	50.0%	50.0%	0.0%	0.0%	0.0%	-	-
Education level	Primary school and below	15.6%	28.9%	53.1%	2.3%	0.0%	-	-
	Middle school (junior high school, senior high school)	23.1%	0.0%	73.1%	3.8%	0.0%	-	-
	University (undergraduate, junior college)	9.5%	25.7%	58.1%	5.4%	1.4%	-	-
	Postgraduate and above	0.0%	0.0%	100.0%	0.0%	0.0%	-	-
Average annual income	<3000	46.4%	53.6%	0.0%	0.0%	0.0%	-	-
	3000–6000	33.3%	50.0%	16.7%	0.0%	0.0%	-	-
	6001–12,000	13.8%	31.0%	55.2%	0.0%	0.0%	-	-
	12,001–24,000	3.6%	16.1%	78.6%	1.8%	0.0%	-	-
	24,001–36,000	13.6%	13.6%	68.2%	4.5%	0.0%	-	-
	36,001–48,000	10.0%	30.0%	50.0%	6.7%	3.3%	-	-
	48,001–60,000	11.1%	22.2%	44.4%	22.2%	0.0%	-	-
	60,001–80,000	0.0%	50.0%	50.0%	0.0%	0.0%	-	-
Degree of understanding	Know very well	11.7%	18.3%	66.7%	3.3%	0.0%	-	-
	General understanding	10.0%	22.0%	62.0%	6.0%	0.0%	-	-
	Do not understand	22.9%	32.3%	42.7%	1.0%	1.0%	-	-
Protection Necessity	Essential	14.0%	25.2%	56.8%	3.6%	0.4%	2.289 (0.512)	0.033 *
	General	66.7%	16.7%	16.7%	0.0%	0.0%	-	-

Note: Total N = 256, Significance levels: *** *p* < 0.001, ** *p* < 0.01, * *p* < 0.05. To highlight the key points of the data presentation, indicators with insufficient valid samples—such as “>80,000” in Average annual income and “No need” in Protection Necessity—have been removed from the analysis.

**Table 4:** Logistic regression analysis of WTP determinants.

Variable	Regression Coefficient	SE	Test Value	Sig.	95% CI
Constants	−9.641	2.019	22.805	0.000 ***	[0.100, 0.610]
Gender	−0.592	0.400	2.193	0.002 **	[0.253, 1.211]
Age	0.043	0.199	0.046	0.001 ***	[0.706, 1.543]
Education level	−0.451	0.279	2.616	0.487	[0.369, 1.100]
Average annual income	−0.105	0.141	0.547	0.220	[0.683, 1.188]
Degree of understanding	1.780	0.708	6.319	0.665	[1.480, 23.755]
Protection Necessity	2.289	0.512	20.016	0.033 *	[3.619, 26.895]
Inspection index	Variance	Freedom	Mean square deviation	Observation value = 326.000	
Residual model	24.829	9.000	2.759	F (9, 315) = 29.435, *p* < 0.001	
	29.522	315.000	0.094	Fitting degree of regression model = 0.457	
Total	54.351	324.000	2.853	R^2^ = 0.892	

Note: Significance levels: *** *p* < 0.001, ** *p* < 0.01, * *p* < 0.05. Variables not included in the regression model (e.g., ethnicity, religion) showed descriptive differences but lacked statistical significance (*p* > 0.05) when controlling for covariates. Y = −9.641 − 0.592 × gender + 0.043 × age − 0.451 × education level − 0.105 × average annual income + 1.780 × degree of understanding + 2.289 × are necessary to protect. While gender and age showed statistically significant associations with WTP (*p* < 0.05), their odds ratio confidence intervals included 1, suggesting these effects, while reliable, may be modest in magnitude. This pattern can occur when (1) effects are small but consistent across the sample, (2) subgroups have varying response patterns that widen the confidence intervals, or (3) continuous variables show non-linear relationships that are captured by the regression but not fully reflected in the point estimates. The significant *p*-values indicate these variables do meaningfully contribute to predicting WTP, while the confidence intervals suggest caution in interpreting the exact strength of these relationships.

**Table 5:** Cumulative frequency distribution of sample average WTP.

$WP_{a_{i}}$/Yuan·a^−1^	10	50	100	200	300	400	500	600	800	>1000	Total
*N_i_*/Person	1	38	64	49	65	8	21	3	5	2	256
$\frac{n_{i}}{N}$/%	0.39	14.84	25.00	19.14	25.39	3.13	8.20	1.17	1.95	0.78	78.77

## Data Availability

The original contributions presented in this study are included in the article. Further inquiries can be directed to the corresponding author.

## References

[1] Acreman M.C., Harding R.J., Lloyd C., Mcnamara N.P., Mountford J.O., Mould D.J., Purse B.V., Heard M.S., Stratford C.J., Dury S.J. (2011). Trade-off in ecosystem services of the Somerset Levels and Moors wetlands. Hydrol. Sci. J..

[2] Buckton S. (2018). Products from Wetlands, Overview.

[3] Ton L.A.N., Smith R.K., Sevilla J. (2024). Symbolically simple: How simple packaging design influences willingness to pay for consumable products. J. Mark..

[4] Arthington A.H., Godfrey P.C., Pearson R.G., Karim F., Wallace J. (2015). Biodiversity values of remnant freshwater floodplain lagoons in agricultural catchments, evidence for fish of the Wet Tropics bioregion, northern Australia. Aquat. Conserv. Mar. Freshw. Ecosyst..

[5] Cockerton H.E., Street-Perrott F.A., Barker P.A., Leng M.J., Sloane H.J., Ficken K.J. (2015). Orbital forcing of glacial/interglacial variations in chemical weathering and silicon cycling within the upper White Nile basin, East Africa, Stable-isotope and biomarker evidence from Lakes Victoria and Edward. Quat. Sci. Rev..

[6] Randler C., Vanhöfen J., Härtel T., Neunhoeffer F., Engeser C., Fischer C. (2023). Psychological restoration depends on curiosity, motivation, and species richness during a guided bird walk in a suburban blue space. Front. Psychol..

[7] Shehawy Y.M., Agag G., Alamoudi H.O., Alharthi M.D., Brown A., Labben T.G., Abdelmoety Z.H. (2024). Cross-national differences in consumers’ willingness to pay (WTP) more for green hotels. J. Retail. Consum. Serv..

[8] Guzy J. (2010). Maintaining Biodiversity with a Mosaic of Wetlands: Factors Affecting Amphibian Species Richness Among Small Isolated Wetlands in Central Florida.

[9] Durgun S., Davras Ö. (2024). Determining the antecedents influences on travel intention and willingness to pay during the pandemic. Int. J. Hosp. Tour. Adm..

[10] Fournier A.M.V., Lancaster J.D., Yetter A.P., Hine C.S., Hagy H.M. (2021). Nest success and nest site selection of wetland birds in a restored wetland system. Avian Conserv. Ecol..

[11] Brandis K.J., Mazumder D., Gadd P., Ji B., Kingsford R.T., Ramp D. (2021). Using feathers to map continental-scale movements of water birds and wetland importance. Conserv. Lett..

[12] Chowdhury S. (2020). Migratory Wetland Birds Diversity in Lower Chota Nagpur Plateau with Special Reference to Purulia District, West Bengal, India. Int. J. Adv. Res..

[13] Sitko J., Heneberg P. (2020). Systemic collapse of a host-parasite trematode network associated with wetland birds in Europe. Parasitol. Res..

[14] Wang S., Xu D., Song S., Shi M., Hu S. (2022). Landscape quality evaluation of national wetland parks based on ecosystem service functions: A case study of the Greater Xing’an Mountains region in Heilongjiang Province. J. Cent. South Univ. For. Technol..

[15] Collop C. (2017). Impact of Human Disturbance on Coastal Birds, Population Consequences Derived from Behavioural Responses. Ph.D. Thesis.

[16] Gu Y., Dong L., Liu Z., Chen Y., Wang T. (2022). Dynamic changes and influencing factors of forest landscape pattern in Maoershan Forest Farm in the past 40 years. J. Cent. South Univ. For. Technol..

[17] Allport G.-A., O’Brien M., Cadbury C.J. (1986). Survey of Redshank and Other Breeding Birds on Saltmarshes in Brita in 1985.

[18] Ferreira N., Lins L., Fink D., Kelling S., Wood C., Freire J., Silva C. (2011). BirdVis, Visualizing and Understanding Bird Populations. IEEE Trans. Vis. Comput. Graph..

[19] Fuller R.J. (1981). The breeding habitats of waders on North Uist machair. Scott. Birds.

[20] Zhao K., He S., Li W. (2010). The Study of Wetland Biodiversity in China. J. Chin. Acad. Sci..

[21] Wang Y., Fu Y.C. (2022). Improved Index Weighting Method for Dynamic Comprehensive Evaluation of Water Resources Carrying Capacity. J. Stat. Inf..

[22] Li L., Wang D., Zhong F., Zhu W. (2007). Search on Environmental Influence Factors of Wetland Bird Diversity. Sichuan J. Zool..

[23] Kumar P., Gupta S.K. (2013). Status of wetland birds of Chhilchhila Wildlife Sanctuary, Haryana, India. J. Threat. Taxa.

[24] Wang R., Li D., Zeng Z., Gao Y., Zhang S., Xiao W. (2021). Diversity and Distribution of Waterbirds in Different Wetland Groups of the Yunnan-Guizhou Plateau. Sichuan Zool..

[25] Zhou C., Brita M.S., Yan J., Xun C., Li K. (2014). Ecological Compensation, A Key to Sustainable Development in the Guizhou Province Karst Region, Southwest China. Open J. For..

[26] Xie G., Lu C., Xiao Y., Zheng D. (2003). Service value evaluation of Alpine Grassland Ecosystem in the Qinghai Tibet Plateau. Mt. Res..

[27] Shi X., Zhao S., Lu S., Wang T., Xu X. (2024). The Effect of Farmers’ Livelihood Capital on Non-Agricultural Income Based on the Regulatory Effect of Returning Farmland to Forests: A Case Study of Qingyuan Manchu Autonomous County in China. Small-Scale For..

[28] Liang X. (2018). Research on Wetland Ecological Compensation Value Measurement Based on Environmental Replacement Cost Method. Master’s Thesis.

[29] Liang C., Liu X., Wang G., Cheng G. (2015). Valuation on Gahai Wetland Ecosystem Service in Gansu. J. Minzu Univ. China Nat. Sci..

[30] Tian J., Ma P. (2002). Plateau Pearl—Gahai—Zecha National Nature Reserve. For. Gansu.

[31] Statistics Bureau of Gannan Tibetan Autonomous Prefecture (2022). Gannan Statistical Yearbook (2021).

[32] Wei L., Ran J., Zhang H., Zhao C., Zhang M. (2014). Distribution of Black-necked Cranes in Sichuan Province. Collection of Abstracts of Papers of the Third China West China Zoological Symposium. Zool. Res..

[33] Liu B., Zhang Y., Wu B., Wu X., Qin S., Zhang J. (2015). Evaluation on the conservation value of animal species diversity in desert ecosystem of China. Sci. Soil Water Conserv..

[34] He C., Ishikawa T., Sheng L., Irie M. (2009). Study on The Hydrological Conditions for the Conservation of the Nesting Habitat of the Red-crowned Crane in Xianghai wetlands, China. Hydrol. Process..

[35] Wang Y.F., Hong C.K., Liu H., Peng F.Y. (2023). Quality Evaluation of Dual-Circulation and Enable Effect of Digital Economy. J. Stat. Inf..

[36] Chen H. (2006). Study on Crane Entertainment and Cultural Value Evaluation in Zhalong National Nature Reserve.

[37] Wang Y., Wang Y., Li S., Sun R. (2011). Evaluation of ecological service function of Futian Mangroves and Birds National Nature Reserve, Shenzhen. J. S. China Norm. Univ. Nat. Sci..

[38] Shi X., Zhang J., Lu J., Zhao T., Yang H., Aria A., Qiu Y., Yu L., Ni Y. (2024). Global Trends and Innovations in Forest Ecological Compensation: An Interdisciplinary Analysis. Forests.

[39] Zhou X., Wang L., Tian Q., Yan Y., Zhou X., Zhao A., Wang L. (2020). Analysis of Bird Fauna and Biodiversity in Gahai Wetland. Gansu For. Sci. Technol..

[40] Ruckelshaus M.H., Jackson S.T., Mooney H.A., Jacobs K.L., Ouyang Z. (2020). The ipbes global assessment: Pathways to action. Trends Ecol. Evol..

[41] Gardella P., Krute L. (2024). Wings of the Gods: Birds in the World’s Religions.

[42] De Yampert R. (2024). Crows and Ravens: Mystery, Myth, and Magic of Sacred Corvids.

[43] Crenshaw J.L. (2025). Monotheism and Wisdom in the Hebrew Bible: An Uneasy Pair? Elements in Religion and Monotheism.

[44] Dunne P. (2024). The Courage of Birds: And the Often Surprising Ways They Survive Winter.

[45] Chen C., Liu X., Yan L., Wang J., Peng P. (2018). Evaluation of ecosystem service value of Nanhe National Wetland Park in Sichuan Province. Wetl. Sci..

[46] Pascual U., Balvanera P., Christie M., Baptiste B., González-Jiménez D., Anderson C.B., Athayde S., Chaplin-Kramer R., Jacobs S., Kelemen E., IPBES (2022). Summary for Policymakers of the Methodological Assessment Report on the Diverse Values Valuation of Nature of the Intergovernmental Science-Policy Platform on Biodiversity Ecosystem Services.

[47] Dethlefsen V., Jackson T., Taylor P. (2024). The precautionary principle—Towards anticipatory environmental management. Clean Production Strategies Developing Preventive Environmental Management in the Industrial Economy.

[48] Martínez-Falcó J., Sánchez-García E., Marco-Lajara B., Lee K. (2024). Green intellectual capital and environmental performance: Identifying the pivotal role of green ambidexterity innovation and top management environmental awareness. J. Intellect. Cap..

[49] Aftab J., Abid N., Sarwar H., Amin A., Abedini M., Veneziani M. (2024). Does corporate social responsibility drive financial performance? Exploring the significance of green innovation, green dynamic capabilities, and perceived environmental volatility. Corp. Soc. Responsib. Environ. Manag..

[50] Liu W., Li J., Li J. (2013). Wild Animal and Plant Resources of the Internationally Important Wetland in Gahai, Gansu. Healthy Lakes and Beautiful China—China Lake Forum and Hubei Science and Technology Forum. https://xueshu.baidu.com/usercenter/paper/show?paperid=0462bf06669e70aa4cd4fb475ff65480&site=xueshu_se.

[51] Shu M. (2017). Research on Reproductive Ecology and Migration of Black-Necked Cranes in Yanchi Bay, Gansu.

[52] Wei W., Li T., Li J. (2010). Protection and management of wetland ecosystem in Gahai. Wetl. Sci. Manag..

[53] Czajkowski M., Zawojska E., Meade N., da Motta R.S., Welsh M., Ortiz R.A. (2024). On the inference about a willingness-to-pay distribution using contingent valuation data. Ecol. Econ..

[54] Albaladejo-García J.A., Pleite F.M.C., Martínez-Paz J.M. (2025). Co-creation of economic value in peri-urban protected areas: Insights from nature conservation and nature-based recreation. Habitat Int..

[55] Alsharari N.M., Aljohani M.S. (2024). The benchmarking implementation and management control process as influenced by interplay of environmental and cultural factors: Institutional and contingency perspectives. Benchmarking Int. J..

